# Comprehensive Overview of Computational Tools for Alternative Splicing Analysis

**DOI:** 10.1002/wrna.70030

**Published:** 2025-12-01

**Authors:** Hieu Tran, Nirad Banskota, Myriam Gorospe, Supriyo De

**Affiliations:** ^1^ Computational Biology and Genomics Core, Laboratory of Genetics and Genomics National Institute on Aging, National Institutes of Health Baltimore Maryland USA; ^2^ RNA Regulation Section, Laboratory of Genetics and Genomics National Institute on Aging, National Institutes of Health Baltimore Maryland USA

**Keywords:** computational analysis of splicing, short‐ and long‐read sequencing, transcriptomic analysis

## Abstract

Alternative splicing (AS) is a fundamental mechanism that generates transcriptomic diversity by selectively including or excluding exons and introns from pre‐mRNA transcripts, leading to the production of multiple protein isoforms from a single gene. This process plays a crucial role in cellular differentiation, tissue specificity, and response to environmental stimuli. Given that it enables organisms to adapt to varying conditions and maintain homeostasis, AS has become a pivotal area of study in molecular biology. The advancement of RNA‐sequencing (RNA‐seq) technologies has propelled the development of sophisticated tools designed to detect and analyze various AS events. These tools have become indispensable for researchers seeking to unravel the complexities of AS and its implications in health and disease. In this review, we delve into the prominent alternative splicing analysis tools rMATS, SUPPA2, LeafCutter, MISO, DEXSeq, MAJIQ, StringTie, and Cufflinks, discussing their strengths, limitations, and practical usability. Each of these tools offers unique functionalities tailored to different aspects of AS analysis, and their usefulness varies depending on computational requirements, ease of use, and the specificity of the AS events they detect. Through careful consideration of the functionalities and limitations of these tools, we offer insights into the biological contexts for which they might be best suited for AS analysis.

This article is categorized under:

RNA Methods > RNA Analyses In Vitro and In Silico
RNA Processing > Splicing Regulation/Alternative Splicing

## Graphical abstract

Through alternative splicing (AS), the cell can generate multiple proteins from a single gene, enhancing proteomic diversity, which vitally supports cellular function and adaptation. This Overview examines key AS analysis tools, including rMATS, SUPPA2, LeafCutter, and others, highlighting their unique features, strengths, and limitations to guide their optimal use in diverse biological contexts.

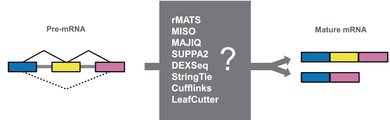

## 1 Introduction

Alternative splicing (AS) is a complex and versatile process in eukaryotic cells whereby different combinations of exons in pre‐mRNAs are spliced together, resulting in the production of various protein isoforms from a single gene. This mechanism robustly increases protein diversity in eukaryotes, despite a limited number of genes. In humans, approximately 95% of multi‐exon genes undergo alternative splicing (Leoni et al. 2011; Pan et al. 2008). Given that the human genome contains around 20,000–25,000 protein‐coding genes, AS plays a vital role in expanding the proteomic repertoire in humans and other organisms (Corbett 2018).

**FIGURE 1:**
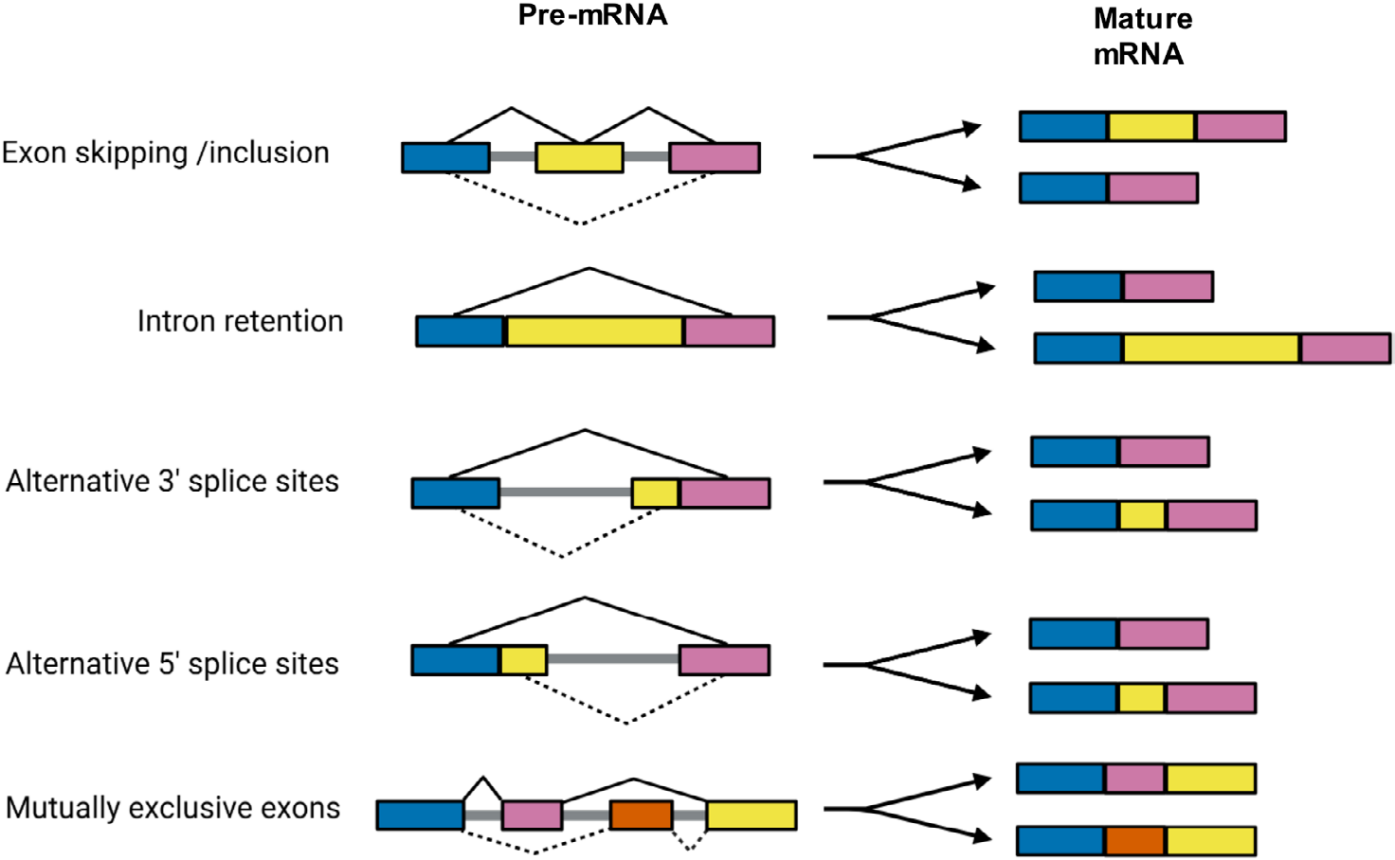
Schematic overview of common alternative splicing mechanisms. The pre‐mRNA transcript undergoes different splicing patterns, resulting in two possible mature mRNA isoforms. Events in the illustration include exon skipping/inclusion, intron retention, alternative 3′ splice sites, alternative 5′ splice sites, and mutually exclusive exons. These splicing variations contribute to transcript diversity and gene regulation. Created using BioRender.

Common AS event types include exon skipping (cassette exons), intron retention, alternative splice sites at the 5′ and 3′ positions, and mutually exclusive exons (Figure 1). These events contribute substantially to protein diversity, impacting cellular processes, development, and adaptation. Studying AS is essential because it profoundly impacts transcriptomic diversity and proteome complexity, enabling normal cellular functions. Conversely, aberrant splicing patterns can lead to dysfunctional protein products, contributing to conditions like Alzheimer's disease (AD), Parkinson's disease, and cancer. For example, in AD, AS events such as exon 8 exclusion in Amyloid‐beta (Aβ) Precursor Protein‐binding Family B, Member 2 (APBB2), can affect Aβ production, a key component of abnormal protein deposits in the brain; similarly, exon 10 inclusion/exclusion in Microtubule‐Associated Protein Tau (MAPT) can alter tau isoform ratios, contributing to neurofibrillary tangle formation, which disrupts neural communication and leads to neuron loss (Love et al. 2015). AS research offers promising insights for personalized medicine by identifying individual‐specific splicing profiles, facilitating biomarker discovery for disease diagnosis and prognosis, and providing targets for therapeutic interventions aimed at modulating splicing outcomes (Liu et al. 2022).

AS also plays a role in cancer and intersects with aging, impacting cellular function and disease processes. Aging can alter AS patterns, leading to changes in protein isoforms and cellular functions. These age‐related AS modulations can contribute to cellular senescence, altered signaling pathways, and impaired genomic stability, all hallmarks of aging cells. The link between AS, aging, and cancer is evident when considering how AS dysregulation in aging cells can promote oncogenic transformations. Age‐related changes in AS may produce aberrant protein isoforms that drive tumor initiation, progression, and metastasis. AS alterations can also modify cellular responses to stress, DNA damage repair mechanisms, and immune surveillance, further influencing cancer development in aging individuals. Understanding the interplay between AS, aging, and cancer is crucial for unraveling the molecular mechanisms underlying tumorigenesis in aging populations. It emphasizes the importance of studying AS dynamics throughout aging and its implications for cancer susceptibility, progression, and therapeutic interventions tailored to age‐related AS patterns (Zhang et al. 2021).

In precision medicine, AS analysis is a valuable tool for guiding personalized treatment strategies. By understanding complex AS patterns and their consequences, healthcare providers can customize therapies for each patient based on their specific AS profiles. One such example is using antisense oligonucleotides (ASOs) to regulate AS and induce exon skipping in Duchenne muscular dystrophy (Le et al. 2015). Another notable example is the development of the first FDA‐approved drug, intronic splicing silencer N1 (ISS‐N1), for the treatment of Spinal Muscular Atrophy (SMA). This ASO‐based therapy prevents the skipping of exon 7 in the *SMN2* gene, thereby restoring functional SMN protein production (Ottesen 2017). These examples highlight the significant impact of AS therapies in customized treatments.

Computational tools are essential for studying AS by facilitating the detection, quantification, and analysis of AS events in high‐throughput RNA‐seq data. These tools use algorithms and statistical methods to identify differentially spliced transcripts, characterize splicing patterns, and quantify isoform expression levels. This capability allows researchers to comprehensively study AS dynamics across different conditions, providing insights into the complexity of AS regulation and its impact on gene expression. Understanding these regulatory mechanisms is crucial for uncovering the role of AS in cellular processes and disease pathogenesis. In this study, we aim to evaluate the performance of various computational tools for AS analysis.

## 2 Tools: Background and Applications

The tools reviewed in this manuscript are widely used in the field and have been previously evaluated for accuracy and scalability in several comprehensive benchmarking studies (Mehmood et al. 2020; Muller et al. 2021; Jiang et al. 2023). The earlier dedicated studies provide valuable context for understanding the strengths and limitations of each of the differential splicing tools, especially in regard to consistency of detection, computational demands, and event‐level performance.

Among the many tools available, we focus on those that are most widely used in the field. While our review criteria include computational performance, accuracy, precision, and the ability to detect specific AS events, we also incorporate findings from prior benchmarking efforts to contextualize their practical value. Our primary emphasis is on practical usability from a real‐world user's perspective, with the intention of serving as a guide for students, researchers, and analysts who need to choose tools that work reliably in diverse environments, not just in idealized academic settings. We pay close attention to features that directly impact usability, such as support for long‐ or short‐read input data, integrated visualization capabilities, ease of graphical user interface (GUIs) for navigation, and installation simplicity. These considerations may be particularly valuable for users who do not have extensive bioinformatics support and need tools that are straightforward to deploy and integrate into existing workflows (Table 1).

**TABLE 1:** Overview of AS tools, including supported analysis, input and output formats, required dependencies, special features, and limitations.

Tool	Availability	Dependencies	Analysis	Input	Output	Special features, limitations, or unique aspects
rMATS	Open source	Python (3.6.12 or 2.7.15) Cython (0.29.21 or 0.29.15 for Python 2) BLAS, LAPACK GNU Scientific Library (GSL 2.5) GCC (>=5.4.0) gfortran (Fortran 77) CMake (3.15.4) PAIRADISE, DARTS, Samtools, STAR (optional)	Quantifies alternative splicing in RNA‐seq replicates. Identifies differential splicing events among groups. Uses a mixed model for splicing levels, uncertainty, and variability. Analyzes splicing changes in detail.	gtf fastq or bam	Counts and describes alternative splicing events by type (e.g., skipped exons, alternative splice sites). Includes RNA details, strand orientation, inclusion/skipping counts, normalized lengths, *p* values, and false discovery rates. Identifies novel splice sites and junctions not in the GTF annotation. Intermediate files contain raw read counts for junctions and exon boundaries. Summary file provides an overview of total and significant splicing events.	Provides rmats2sashimiplot, a tool for creating plots from rMATS output. Creates plots using an annotation file with genomic coordinates or directly from SAM/BAM files. Uses MISO as the plotting backend. Supports different grouping methods for sample group comparisons.
MISO	Open source	Python 2.6 or higher numpy (version > 1.5) scipy pysam (version 0.6 or higher) matplotlib (for sashimi_plot) samtools (for accessing SAM/BAM files) bedtools (optional, for computing overlaps and intersections in BAM/GFF files)	Quantifies alternatively spliced RNA abundance. Identifies differentially regulated isoforms or exons. Uses Bayesian inference for read origin probabilities. Provides estimates, confidence intervals, and differential expression at isoform/exon levels. Supports single‐end and paired‐end RNA‐Seq data. Designed for cluster or distributed computing systems	gff bam	Outputs isoform expression estimates (PSI) with 95% confidence intervals. Includes raw read counts supporting each isoform and assigned read counts per isoform. Provides summary statistics for isoform events, including posterior means, confidence intervals, and Bayes factors for differential expression assessment. Creates tab‐separated summary files with event names, isoform names, raw counts, and inferred read assignments. Outputs Bayes factors files for pairwise comparisons, containing differential expression metrics between samples.	Provides utilities for compressing and storing output in SQLite databases. Includes sashimi_plot for visualizing RNA‐Seq reads along isoforms with MISO estimates. Supports single‐end and paired‐end RNA‐Seq data. Enables exon‐centric and isoform‐centric analyses. Requires GFF3 annotations for alternative splicing events or isoforms. Optimized for parallel computing and cluster environments.
MAJIQ	Academic and Commercial Use	Python (3.8 or later) setuptools (45 or later) HTSlib (1.10 or later) C++ compiler with C++11 support (e.g. gcc >= 4.8.1)	MAJIQ and Voila define, quantify, and visualize local splicing variations (LSV) from RNA‐Seq data.	bam bam.bai gff3	Defines splice graphs and identifies known and novel Local Splice Variations (LSVs). Quantifies relative LSV abundance (PSI) and changes (delta PSI) between conditions, with or without replicates. Outputs results in TSV, modular formats, and interactive visualizations through Voila. Supports various result combinations for comprehensive analysis and visualization.	Voila, MAJIQ's visualizer, converts quantified results into TSV, modular, and interactive formats. Supports datasets with varying read lengths and strands, requiring the longest read length in the settings file. Utilizes multiple cores for parallel processing and is optimized for cluster usage. Requires an INI format configuration file specifying study details, annotation, experiments, and parameters. Uses “half‐exons” for ambiguous exon boundaries, which may appear as “unk” (unknown) in Voila if the reference exon is undefined.
SUPPA2	Open Source	Python (3.4)	Study splicing at the transcript isoform or local alternative splicing event level. Analyze across multiple conditions with high speed and accuracy. SUPPA2 offers various modular operations that can be run separately.	gtf ioe (for local events and can be created with SUPPA2) ioi (for transcript‐level and can be created with SUPPA2)	Creates two main files for differential splicing analysis, 1. dpsi file containing event IDs with ΔPSI values (difference in PSI between conditions) and *p* values for significance. 2. psivec file containing PSI values for each replicate or condition across samples. These outputs enable accurate quantification and comparison of differential splicing events between conditions.	Provides comprehensive functionality for PSI calculation, differential splicing analysis, and clustering of splicing events. Lacks built‐in visualization tools, requiring external tools for visual representation of results.
DEXSeq	Open Source	R Package in Bioconductor	Identifies differential exon usage by comparing exon counts among samples. Assesses statistical significance using dispersion to account for replicate fluctuations. Works directly with count data, unlike ratio‐based methods. Supports complex experimental designs with GLMs for ANOVA‐like analyses.	sam or bam gtf metadata (txt)	Outputs a DEXSeqResults object after testing for differential exon usage. Contains information on each exon within analyzed RNAs, including Exon‐specific counts, dispersion estimates, log2 fold changes, *p* values, and adjusted *p* values. Provides summaries of significant exons and affected RNAs for comprehensive analysis and interpretation of differential exon usage across conditions.	DEXSeq is an R package within Bioconductor with a built‐in visualization function, plotDEXSeq(), for visualizing differential exon usage results. First analysis step requires aligning reads to a reference genome using a splice‐aware aligner (e.g., TopHat2, GSNAP, or STAR), not pseudo‐alignment methods. Requires additional steps to prepare annotations, such as creating a flattened annotation file to consolidate overlapping exons and RNAs for accurate exon counting.
StringTie	Open Source	C++ compiler which supports the C++ 11 standard (GCC 4.8 or newer)	Reconstructs transcript structures and quantifies expression from RNA‐Seq using a network flow algorithm with optional de novo assembly. Processes coordinate‐sorted SAM/BAM/CRAM alignments to recover complete transcript isoforms. Supports short‐read alignments and longer assembled sequences. Outputs compatible with Ballgown, Cuffdiff, DESeq2, and edgeR for differential analysis of RNA abundance.	sam/bam/cram gtf or gff3 fasta (optional for cram files)	GTF output includes assembled transcripts with exon boundaries, start and end points, and RNA IDs. Expression estimates include FPKM and TPM values for transcripts and RNAs. RNA abundance file contains coverage, FPKM, and TPM per RNA. The ‐C option generates a GTF file of fully covered reference transcripts. The ‐B option produces files for Ballgown differential expression analysis. Merge mode creates a non‐redundant merged GTF for consistent analysis.	Notable limitation of StringTie: lacks a built‐in visualization feature, requiring users to rely on external tools.
Cufflinks	Open Source	Boost C++ libraries (1.47 or later)	Cufflinks is a suite of tools for RNA‐Seq data analysis. Assembles RNA‐Seq reads into transcripts. Estimates transcript abundances. Tests for differential abundance levels.	sam or bam gtf or gff	Outputs assembled transcripts with estimated abundances, differential expression results, and regulation information. Generates transcript and RNA‐level expression data for downstream analyses like clustering, visualization, and statistical comparisons across samples.	Paired‐end sequencing improves transcript assembly and expression accuracy. Lacks built‐in visualization tools, requiring external tools like Plotly.
LeafCutter	Open Source	samtools should be available on your PATH regtools should be available on your PATH Python 2.7 (earlier versions may be OK) R (version 3.6.0, earlier versions may be OK)	Quantifies RNA splicing variation using short‐read RNA‐seq data. Leverages spliced reads to quantify intron usage across samples.	fasta bam to junc	Easily detects novel introns. Models more complex splicing events than exonic PSI. Avoids the challenge of isoform abundance estimation. Utilizes simple, computationally efficient algorithms that scale to hundreds or thousands of samples.	Outdated software results in installation difficulties. One possible solution is creating a container from a successful installation.

Previous benchmarking efforts have shown wide variability in detection counts, precision, and resource demands across AS tools. For example, SUPPA2 and Cufflinks were found to produce relatively low numbers of detected splicing events, while tools like rMATS and MAJIQ showed more consistent detection across sample sizes and splicing types (Mehmood et al. 2020; Jiang et al. 2023). Muller et al. further emphasized the practical implications of these differences, noting that tools like rMATS and SUPPA2 maintained low CPU and RAM usage even on low‐resource systems, while MISO consistently exceeded resource limits across virtual machine configurations (Muller et al. 2021). Our own analysis confirms these trends but emphasizes how they translate into practical usability for everyday users.

### 2.1 rMATS

Replicate Multivariate Analysis of Transcript Splicing (rMATS) is a method developed by Yi Xing's team, building upon its predecessor, MATS (Shen et al. 2014). The primary function of rMATS is to detect differential AS between replicate RNA‐seq datasets. Compared to other methods for analyzing AS in RNA‐seq data, rMATS has several notable features.

Since rMATS (and its accelerated variant, rMATS‐turbo) is optimized for processing short‐read RNA‐seq data (Shen et al. 2014), researchers aiming to analyze long‐read RNA‐seq data should instead employ rMATS‐long, an integrated workflow explicitly designed for long‐read analyses. rMATS‐long leverages the novel capabilities of ESPRESSO, a tool developed for accurate transcript discovery and quantification from error‐prone long‐read sequences (Gao et al. 2023). While short reads offer high‐throughput data for known AS events, which is useful for initial screenings, long reads span entire transcripts, providing a more accurate representation of transcript isoforms and permitting more precise quantification of splicing events, as well as the identification of novel splice variants. rMATS employs a hierarchical model that combines binomial and normal distributions for unpaired replicates and a bivariate normal distribution for paired replicates. This model includes length normalization functions and utilizes likelihood‐ratio tests for hypothesis testing. It also allows for the assessment of statistical significance based on user‐defined splicing change magnitudes, providing a flexible approach to differential splicing analysis.

rMATS has been shown to scale well with increasing sample sizes and maintain stable precision and recall. These features make it a dependable choice for users working with larger datasets or variable replicate quality. Our own runtime and memory analysis (below) supports its efficiency and consistency (Mehmood et al. 2020). Although retained intron (RI) event detection showed lower correlation between rMATS and other tools (Muller et al. 2021), rMATS maintained low CPU and RAM usage across all virtual machine configurations, even under increasing dataset sizes, making it suitable for low‐resource environments.

Xing and colleagues compared the performance of rMATS to that of two other differential splicing tools, Cufflinks and DiffSplice. They found that these methods differ in their handling of data points with varying read counts and estimation uncertainties. rMATS gives less weight to replicates with low read counts, unlike Cufflinks and DiffSplice, which treat all replicates equally (Hu et al. 2013). Simulations using TCGA clear cell renal carcinoma (ccRCC) RNA‐seq data and receiver operating characteristic (ROC) curves showed that rMATS had better performance, with an area under the curve (AUC) of 86%, compared to 83% for Cufflinks and 81% for DiffSplice. Additionally, in regions where false positive rates were less than 0.2, rMATS achieved true positive rates (TPRs) of up to 8% and 15% higher than Cufflinks and DiffSplice, respectively. Additional tests introduced replicates with low read coverage or high variability, further underscoring the strength of rMATS' performance. In these two scenarios, when false positive rates were below 0.2, rMATS improved its true positive rates by up to 19% and 16% over both Cufflinks and DiffSplice, respectively. Overall, rMATS consistently outperformed Cufflinks and DiffSplice, particularly by better accounting for the uncertainty in isoform proportion estimates (Shen et al. 2014).

### 2.2 MISO

MISO (Mixture‐of‐Isoforms) is a probabilistic tool designed by Yarden Katz and coworkers to analyze RNA‐seq data (Katz et al. 2010). It quantifies the expression levels of alternatively spliced transcripts and identifies differentially abundant isoforms or exons. MISO offers robust statistical modeling and high validation rates, although it consumes more memory and CPU resources than other tools. Users should consider this factor when working on shared clusters or with constrained compute environments (Mehmood et al. 2020; Muller et al. 2021).

MISO models how RNA‐seq reads are generated from isoforms and utilizes Bayesian inference to assign probabilities to reads originating from specific isoforms. This approach enables the estimation of isoform expression levels (Percent Spliced In (PSI) or Ψ values) through a Markov Chain Monte Carlo (MCMC) sampling method. MISO provides confidence intervals and Bayes factors (BFs) to assess the reliability of expression estimates and measures of differential expression. It supports both exon‐centric and isoform‐centric analyses and is optimized for use in cluster computing environments. It requires minimal dependencies, including Python modules and samtools (Li et al. 2009).

To use MISO, users need to select and index a GFF annotation set, run MISO to obtain expression estimates, summarize the results, and perform pairwise comparisons to detect differential expression across samples. It is important to note that MISO annotations for events consider only the exons immediately flanking an alternative event. For example, in a skipped exon event, only the two neighboring exons are included, resulting in isoforms roughly 400 nucleotides and 300 nucleotides long. For long paired‐end libraries, where the average insert size is significantly longer than these isoforms, it may be more appropriate to use modified annotations that incorporate more exons.

Simulations show that MISO provides lower variance and error in splicing estimates compared to standard splice‐junction estimates. MISO's credible intervals help identify more reliable splicing events, and its use of the BF for differential splicing analysis offers a robust method for detecting differentially expressed isoforms. Additionally, MISO's estimates show high rates of validation using reverse transcription followed by quantitative polymerase chain reaction (RT‐qPCR) analysis, indicating high accuracy.

MISO is applicable to genome‐wide studies and effectively identifies and validates splicing events regulated by factors such as HNRNPH using CLIP‐seq data. It also handles alternative polyadenylation (APA) events and employs probabilistic read assignment to improve isoform abundance estimation, enhancing the overall accuracy and reliability of RNA processing event analyses (Katz et al. 2010).

### 2.3 MAJIQ

MAJIQ and Voila are software packages designed to define, quantify, and visualize local splicing variations (LSVs) from RNA‐seq data. MAJIQ has demonstrated consistent detection counts across sample sizes and relatively low memory usage. It also maintains a low false discovery rate (FDR), making it a practical option for users who prioritize reproducibility and computational efficiency (Mehmood et al. 2020). MAJIQ consists of three main modules: MAJIQ Builder, MAJIQ Quantifier, and Voila (Vaquero‐Garcia et al. 2023). MAJIQ Builder processes RNA‐seq BAM files and a transcriptome annotation file to define splice graphs, identifying both known and novel LSVs. MAJIQ Quantifier then quantifies the relative abundance (PSI) of LSVs and calculates changes in relative LSV abundance (delta PSI) between conditions. Voila converts these quantified results into human‐usable outputs, offering formats such as TSV, modularized reports, and interactive visualizations.

Before using MAJIQ, sequences must be processed and their quality checked to create BAM files along with their indices. Additionally, a gene annotation database in GFF3 format is required. The MAJIQ v2 pipeline supports RNA splicing analysis using large RNA‐seq datasets (Vaquero‐Garcia et al. 2023). In this pipeline, the builder module combines transcript annotations and coverage from aligned RNA‐seq experiments to construct updated splice graphs. The quantifier module then employs Bayesian models to estimate inclusion levels (PSI) and changes between conditions (dPSI). Voila v2 enhances visualization capabilities, allowing users to filter quantified PSI and dPSI results, and supports sharing these results with collaborators.

MAJIQ is compatible with both short and long reads, and the MAJIQ‐L extension provides a unified view of transcriptome variations from both technologies. Short reads offer better coverage and lower error rates, while long reads capture entire isoforms and can identify more intron retention events. Combining both types of reads is beneficial for comprehensive transcriptome analysis, leveraging the strengths of each to provide a more complete and accurate representation of splicing events (Han et al. 2023).

According to studies conducted by Jorge Vaquero‐Garcia et al. MAJIQ v2 demonstrates superior performance in various aspects compared to other AS analysis tools such as rMATS, LeafCutter, SUPPA2, and Whippet (Sterne‐Weiler et al. 2018). While SUPPA2 is faster when parallelized, it suffers from a higher false discovery rate (FDR) of around 15%–30% and a high false negative rate (FNR) of 49%. In contrast, MAJIQ maintains a significantly lower FDR of 0.3% and has an FNR comparable to that of LeafCutter, which ranges from 2.5% to 5.5% depending on the sample size (Vaquero‐Garcia et al. 2023). MAJIQ and its extended version, MAJIQ HET, report a higher number of differentially spliced genes and events with greater resolution. For example, MAJIQ identifies 34% more changing RNA splice variants and 6% more non‐changing RNA splice variants than LeafCutter. This increased detection accuracy is crucial for comprehensive AS analysis. The accuracy of MAJIQ is validated through large‐scale synthetic datasets and real Genotype‐Tissue Expression Project (GTEx) data, consistently showing lower intra‐to‐inter ratio (IIR) values, indicating robust performance with a low FDR even in small sample sets.

In reproducibility tests, MAJIQ and MAJIQ HET exhibit high reproducibility ratios across different sample sizes, outperforming other methods. Moreover, MAJIQ's delta PSI (dPSI) accuracy is confirmed through comparisons by RT‐qPCR analysis, achieving a high correlation of approximately 0.97–0.98. This level of accuracy surpasses that of LeafCutter and SUPPA2, further establishing MAJIQ's reliability and precision in detecting and quantifying AS events (Vaquero‐Garcia et al. 2023).

### 2.4 SUPPA2

SUPPA2 is an open‐source tool for differential splicing analysis developed by Eduardo Eyras and team, designed to study AS at both the transcript isoform level and the local splicing event level (Trincado et al. 2018). SUPPA2 is exceptionally fast and lightweight, completing analyses in minutes and using minimal memory at small sample sizes. However, users wishing to scale up should be mindful that its detection counts and FDR can vary more widely with larger datasets (Mehmood et al. 2020). It supports both short and long reads, offering flexibility in its applications. For short reads, SUPPA2 demonstrates a higher rate of true positives compared to other tools. The tool is modular and capable of performing multiple operations independently, including generating transcript and local AS events from annotation inputs, quantifying transcripts and the percent spliced index (PSI) from multiple samples, calculating differential splicing across conditions with replicates, and clustering events and transcripts based on PSI values across conditions.

SUPPA2 analyzes both local AS events and transcript‐level events per gene. Local AS events represent standard splicing variations, while transcript events describe each isoform of a gene separately. These events are generated from an annotation file in GTF format using only the exon lines. SUPPA2 produces different outputs during the event‐generation step, depending on the use case. When generating events at the transcript level, SUPPA2 outputs a single ioi file. This file provides a transcript‐level representation of AS by listing all transcript events within a gene. Each row contains the chromosome name (seqname), gene identifiers, an event identifier formatted as gene_id; transcript_id, the transcript IDs that define the event, and all the transcript IDs for that gene.

Alternatively, for local AS events, SUPPA2 generates an “ioe” file and a GTF file. The ioe file maps each splicing event to the transcripts that define it, including details such as the chromosome name (seqname), gene ID, event ID, and associated transcripts used to calculate PSI values. The GTF file can be loaded into the UCSC Genome Browser for visualization. Eyras et al. compared SUPPA2 to three other methods for calculating differential splicing using multiple replicates per condition: rMATS, MAJIQ, and DEXSeq. They found that SUPPA2 was significantly faster, taking only 24 s for PSI quantification and about 32 min and 47 s for differential splicing analysis on the same datasets. Unlike other methods, SUPPA2 performs the significance test directly on the ΔPSI values without revisiting the read data, providing unmatched speed. Their comparison revealed that rMATS and DEXSeq detect many seemingly significant events with small inclusion changes that are indistinguishable from biological replicate variability. In contrast, SUPPA2 and MAJIQ effectively separate these distributions. By using between‐replicate variability for significance testing, SUPPA2 avoids using an arbitrary global |ΔPSI| threshold, allowing it to detect significant events across a wide range of transcriptome abundance values. This capability helps better rationalize |ΔPSI| threshold cut‐offs (Trincado et al. 2018).

### 2.5 DEXSeq

DEXSeq is a tool developed to quantify differential exon usage in RNA‐seq data using generalized linear models, ensuring reliable control of false discoveries by accounting for biological variation (Anders et al. 2012). DEXSeq is known for its high precision and sensitivity to subtle exon usage changes. Users with time‐sensitive or resource‐constrained workflows should be mindful that DEXSeq requires longer runtimes and higher memory usage (Mehmood et al. 2020). It detects RNA isoforms and exons subject to differential usage, facilitating the study of alternative exon regulation and function on a transcriptome‐wide scale. Available as an R package under Bioconductor, DEXSeq stands out for its focus on exon‐level analysis and its robust statistical approach. Compared to other AS tools like rMATS and SUPPA2, DEXSeq is notable for its ability to detect subtle changes in exon usage. Although DEXSeq was originally developed for short reads, it can also be used for long reads. It employs an exon count‐based strategy to quantify exon levels and compare differential states (Anders et al. 2012). Recent studies have successfully used DEXSeq to detect differential exon usage (DEUs) with long reads, demonstrating its versatility across different sequencing platforms (Leshkowitz et al. 2022).

### 2.6 StringTie

StringTie is a computational method designed for transcriptome assembly that leverages a network flow algorithm from optimization theory, along with optional de novo assembly, to generate more complete and accurate transcript reconstructions compared to other leading tools such as Cufflinks, IsoLasso, Scripture, and Traph (Pertea et al. 2015). Although not included in previous benchmarking studies (like Mehmood et al. 2020), our analysis shows that StringTie is efficient in both runtime and memory usage, especially when combining short and long reads. Its transcript‐level precision and speed make it a strong candidate for users with a particular interest in assembly and quantification.

Developed to handle both short and long reads, StringTie utilizes the –mix option, allowing the first BAM file to be for short reads and the second for long reads. This capability enhances transcriptome assembly by combining data types, resulting in more robust and comprehensive assemblies.

In comparative analyses performed by Mihaela Pertea et al. StringTie demonstrated superior performance. For instance, it assembled 10,990 transcripts from 90 million human blood reads, a 53% increase over Cufflinks. On simulated data, StringTie assembled 7559 transcripts, 20% more than Cufflinks. Additionally, StringTie runs faster than its counterparts across various datasets. While numerous methods address transcript identification (e.g., Trinity, Oases) and expression quantification (e.g., RSEM, eXpress), consistency and accuracy remain challenging. An earlier study (Steijger et al. 2013) highlighted that existing methods often fail to assemble complete isoforms despite identifying all constituent exons, underscoring the need for more precise tools to identify novel transcripts.

StringTie addresses these challenges by simultaneously assembling transcripts and estimating their expression levels. It groups reads into clusters, creates splice graphs, and uses a maximum flow algorithm to estimate expression levels. Unlike Cufflinks, which employs a parsimony‐based approach, StringTie integrates transcript abundance into its assembly process, resulting in higher accuracy. StringTie also uses super‐reads (SR), an assembly approach that extends original short reads by adding bases at both ends until no unique extension is possible. This results in a smaller set of super‐reads that retains all the original sequence information. By utilizing k‐mer counting to identify unique extensions, many original reads can contribute to the same super‐read, reducing the dataset size. These maximal super‐reads are then assembled with other data, such as mate pairs, to form a complete genome assembly (Zimin et al. 2013).

Evaluations using simulated datasets (Sim‐I and Sim‐II) demonstrated that StringTie+SR (which includes super‐read inputs) outperformed other assemblers in terms of sensitivity and precision. For example, StringTie+SR found 20% more true transcripts on Sim‐I with 34% fewer false positives. On Sim‐II, StringTie+SR also outperformed Cufflinks, showcasing its superior sensitivity and precision. The accuracy of StringTie extends to gene‐level identification, consistently showing better sensitivity and precision across various metrics. Moreover, StringTie accurately quantifies transcript expression levels, as evidenced by high Spearman correlation coefficients between true and predicted expression levels. These results highlight the usefulness and precision of StringTie, making it a valuable tool for transcriptome assembly and quantification, especially in complex RNA‐seq datasets (Pertea et al. 2015).

### 2.7 Cufflinks

The software tool Cufflinks was developed through a collaboration among the laboratories of L. Pachter (UC Berkeley), S. Salzberg (Johns Hopkins University), and B. Wold (Caltech) and maintained by C. Trapnell (University of Washington) (Trapnell et al. 2010). Cufflinks is designed to assemble transcripts and estimate their abundance in RNA‐seq samples. From aligned RNA‐seq reads, Cufflinks constructs a set of transcripts, estimates their relative abundances, and accounts for biases in library preparation protocols.

Cufflinks uses a comparative transcriptome assembly algorithm based on maximum matching in a weighted bipartite graph, ensuring minimal assumptions about sequencing experiments. This algorithm recovers both known and novel isoforms, validating transcripts by comparison with existing annotations. Cufflinks estimates transcript abundance using a statistical model that accounts for the probability of observing each fragment as a function of transcript abundance, incorporating fragment length distributions. Abundance estimates are reported in fragments per kilobase of transcript per million fragments mapped (FPKM). The inclusion of novel isoforms significantly impacts abundance estimates, highlighting the importance of integrating transcript discovery with abundance estimation. Cufflinks can identify significant changes in transcript abundance for numerous genes, revealing dynamics not reflected at the gene level. It can also classify expression dynamics into trajectories, grouping transcripts by transcription start sites (TSS) to distinguish between transcriptional and post‐transcriptional regulation. This comprehensive approach allows for a more nuanced understanding of the levels of different RNAs, making Cufflinks a valuable tool for transcriptome analysis (Trapnell et al. 2010).

### 2.8 LeafCutter

LeafCutter, developed by David Knowles and colleagues, is a tool optimized for short‐read RNA‐seq data to detect intron excision events with base‐pair precision by analyzing mapped split reads (Li et al. 2018). It focuses on various alternative splicing events such as skipped exons, alternative splice‐site usage at the 5′ and 3′ ends, and other complex events summarized by differences in intron excision. Unlike isoform‐quantification methods like Cufflinks, LeafCutter does not directly measure alternative transcription start sites or alternative polyadenylation (APA), as these are not generally captured by intron excision events.

The main advantage of LeafCutter's intron‐centric approach is that it bypasses the need for read assembly or isoform inference, which are computationally and statistically challenging. This approach significantly improves speed and memory requirements, outperforming similar methods like MAJIQ. To identify alternatively excised introns, LeafCutter pools all mapped reads from a study and finds overlapping introns identified by split reads. It then constructs a graph that connects all overlapping introns sharing a donor or acceptor splice site. The connected components of the graphs form clusters, representing alternative intron excision events. Finally, LeafCutter applies a filtering step to remove rarely used introns, defined by the proportion of reads supporting a given intron compared with other introns in the same cluster, and re‐clusters the remaining introns. This filtering step helps avoid large clusters at read depths where noisy splicing events are supported by multiple reads.

Yang Li and team conducted a comprehensive comparison of LeafCutter with other differential splicing detection methods, including Cufflinks, MAJIQ, and rMATS. They acknowledged the challenge of comparing algorithms due to the lack of a direct mapping between splicing events quantified by different methods. To assess performance, they applied each method to identify splicing differences across varying sample sizes of Yoruba (YRI) and European (CEU) lymphoblastoid cell line (LCL) RNA‐seq samples. Their findings revealed significant differences in scalability, particularly in runtime. LeafCutter stood out by completing all comparisons within just 1 h, whereas Cufflinks, rMATS, and MAJIQ took substantially longer, up to 7.8, 55.7, and 66.2 h, respectively. Regarding memory usage, LeafCutter used less than 400 MB of RAM for all comparisons, while MAJIQ required over 50 GB for larger comparisons (Li et al. 2018).

## 3 Standardized Review

**FIGURE 2:**
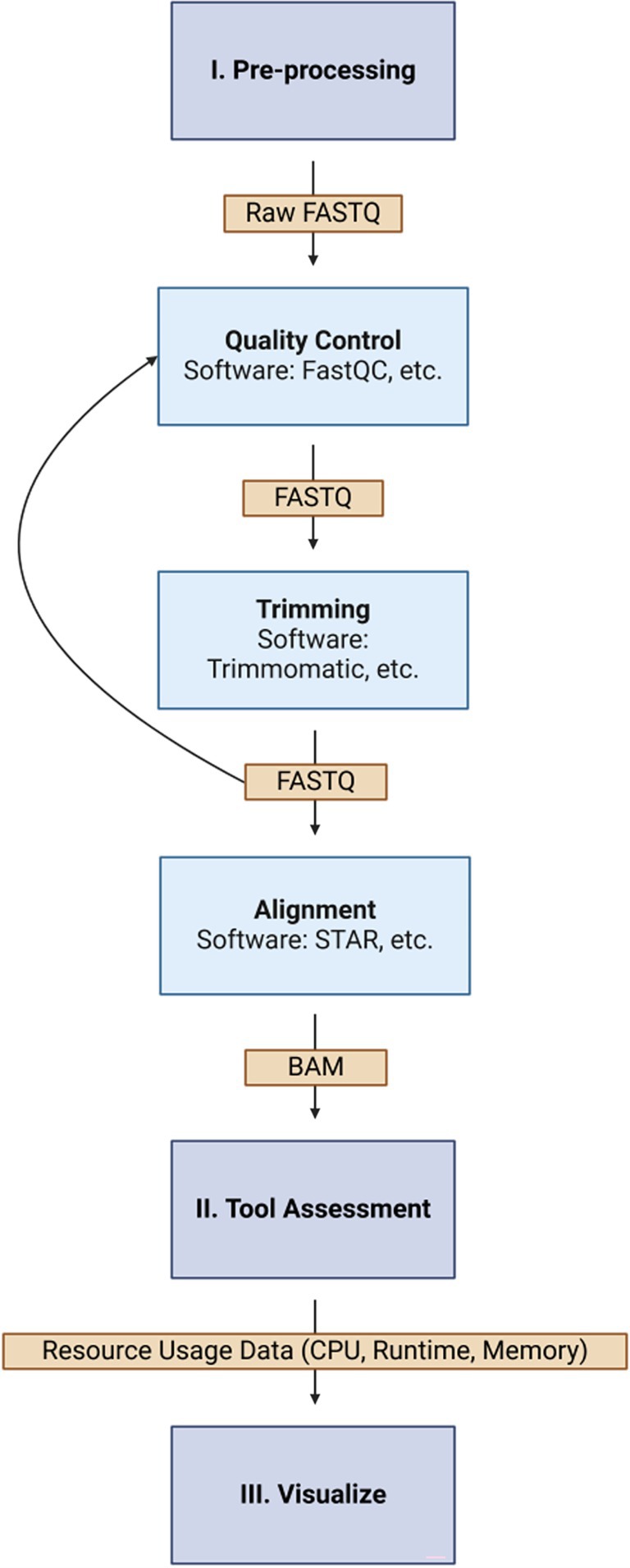
Overview of the general computational steps in RNA‐seq analysis, followed by AS analysis. The workflow illustrates the main steps (blue boxes), their sub‐steps (light blue boxes), and corresponding outputs (orange boxes). The process begins with raw read pre‐processing (quality control and trimming), followed by alignment to a reference genome. The resulting output is then used to evaluate each alternative splicing tool according to its documentation. Finally, the computational resource usage of each tool is recorded and visualized with the goal of providing a user's guide to performing AS analyses across different types of sequencing data. The features considered are intended to evaluate real‐world usability, including ease of installation, compatibility with existing workflows, and clarity of documentation.

For this review, we decided to standardize our comparison by using the same dataset from the “Transcriptome signature of cellular senescence” paper (Casella et al. 2019). This dataset provides a comprehensive overview of the transcriptomic changes associated with cellular senescence, making it an ideal choice for evaluating the performance of various transcriptome analysis tools. By applying each tool to this dataset, we aim to qualitatively assess their accuracy, sensitivity, and efficiency in detecting alternative splicing events, assembling transcripts, and estimating gene and isoform expression levels. Using this standardized dataset will allow us to support our review by directly comparing the outputs, performance metrics, and computational efficiency of the tools (Figure 2).

Like with most downstream analyses, before running any of these alternative splicing tools, we performed quality control (I. Pre‐processing), trimming our reads to remove low‐quality bases and adapter sequences that could interfere with the analysis. This step ensures that only high‐quality data are used, which is especially important for users aiming to minimize troubleshooting during alignment and splicing detection. Proper quality control can improve the accuracy of identifying splicing patterns and isoform quantifications. In this case, we aligned our reads to the reference genome hg38 to map the sequencing reads to their corresponding genomic locations.

We followed the original instructions for each tool, noting where documentation was clear or where additional effort was needed to resolve setup issues. Including this dimension was necessary since each tool's workflow may differ somewhat. For instance, MISO requires reads to be trimmed to a uniform length to avoid errors. Meanwhile, SUPPA2, when paired with Salmon, necessitates the generation of an index based on transcript annotations. Notably, Salmon is designed to quantify transcript expression efficiently by quasi‐mapping RNA‐seq reads to a transcriptome index, which must be created using transcript annotations rather than a genome assembly. This is necessary for tools like SUPPA2 that rely on transcript‐level quantifications for downstream alternative splicing analyses.

Additionally, while preparing to run LeafCutter, we encountered installation difficulties that may be common for users without dedicated IT support, which required verifying dependencies and resolving compatibility issues within our computational environment. Although we were unable to successfully install and include LeafCutter in our comparison, its applications in AS analysis make it an important tool to mention. We considered several potential workarounds to address the installation challenges, one of which involved using Docker container to pull a working instance of LeafCutter from laboratories that have successfully implemented it.

Depending on the categorization of the AS tool, the outputs can vary. Some tools assemble transcripts either *de novo* from sequence reads or by utilizing a reference genome to guide the assembly. Others focus on quantifying isoform expression levels or identifying specific splicing events such as exon skipping, intron retention, or alternative splice site usage. Each tool's unique approach and output format highlight the diverse methodologies employed in alternative splicing analysis, making it essential to choose the right tool based on the specific requirements of the study.

## 4 Assessment of Alternative Splicing Tools

**FIGURE 3:**
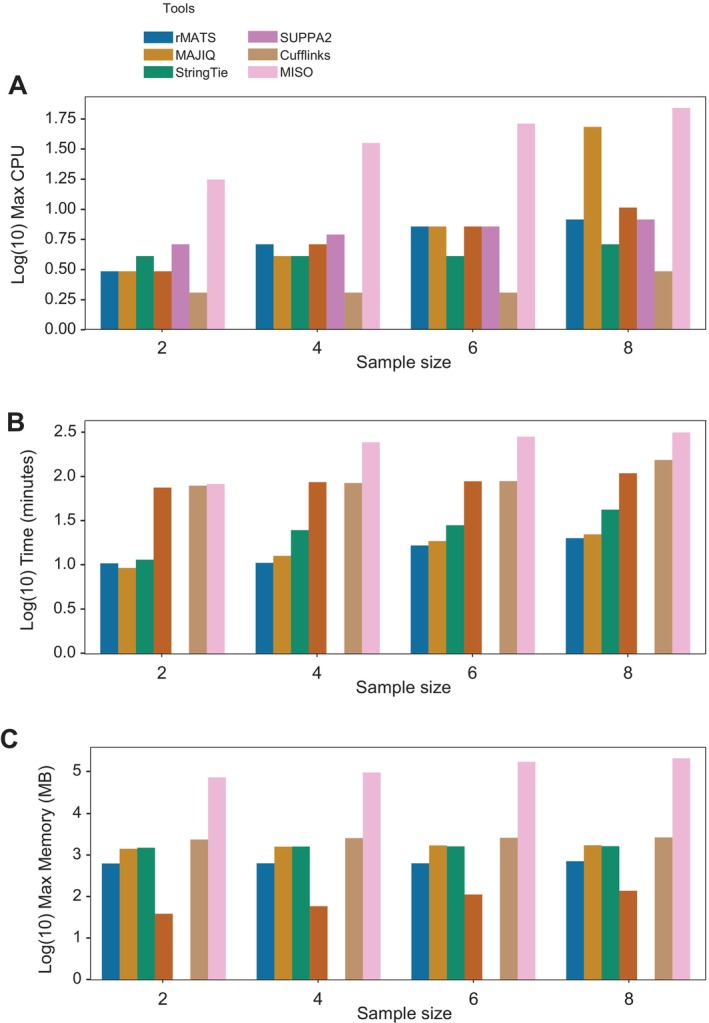
Assessment of the computational resource demands (log10‐transformed) of several alternative splicing quantification tools as sample size increases. The analysis includes sample sizes of 2, 4, 6, and 8, with resource usage visualized for each tool. (A) Maximum CPU usage; (B) total runtime in minutes; (C) maximum memory usage in megabytes. These plots highlight the computational scalability and efficiency of different tools in AS analysis.

We assessed the performance of seven of the eight AS tools mentioned in this paper, excluding LeafCutter due to reasons mentioned above. We tested each tool starting with two samples and increased the sample count in increments of two until we reached eight samples. This approach allowed us to compare the tools' performance as the sample size increased, providing insights into their scalability. The parameters analyzed included runtime, memory usage, and CPU usage (Figure 3). Unlike previous benchmarking efforts (such as Mehmood et al. 2020), our evaluation is designed as a practical guide for users who need to make informed decisions based on real‐world constraints like ease of installation, runtime efficiency, and resource availability.

### 4.1 CPU Usage

To accommodate the wide range of observed values, we applied a log10 transformation. MISO exhibited significantly higher CPU usage compared to the other tools (Figure 3A). Its CPU usage scaled with the sample size, using approximately 16, 33, 48, and 65 CPUs for 2, 4, 6, and 8 samples, respectively. In contrast, most other tools displayed similar CPU usage, remaining relatively low and consistent across all sample sizes.

### 4.2 Runtime

Runtime was evaluated as the total time taken by each tool to process the data as sample sizes increased (Figure 3B). Most tools demonstrated a steady, nearly linear increase in runtime. rMATS, MAJIQ, and StringTie performed efficiently, with runtimes ranging from approximately 8 min to 35 min at larger sample sizes. DEXSeq, Cufflinks, and MISO, however, had significantly longer runtimes, often taking several hours to complete. This was particularly expected for tools like Cufflinks, which focuses on transcript assembly and quantification (Trapnell et al. 2010), a process that can be time‐intensive. In stark contrast, SUPPA2 consistently exhibited the shortest runtime, completing its analyses in 5–10 min regardless of the sample size.

### 4.3 Memory Usage

Memory usage varied widely across the tools (Figure 3C). MISO again stood out, requiring considerably more memory than other tools, with usage scaling from approximately 64,000 MB (64 GB) for 2 samples to 183,000 MB (183 GB) for 8 samples. By comparison, other tools showed relatively stable and lower memory requirements. Notably, SUPPA2 demonstrated exceptional efficiency, using less than 1 MB of memory for 2 samples and around 1 MB for 4, 6, and 8 samples. On the other hand, Cufflinks also displayed low memory usage compared to other tools, requiring only approximately 1000 MB (1 GB) for 2, 4, and 6 samples, and approximately 2000 MB (2 GB) for 8 samples.

## 5 Conclusion

In this review, we evaluated the strengths, limitations, and applications of several alternative splicing (AS) detection tools. We provided an in‐depth comparison of their workflows, input requirements, scalability, and performance benchmarks, including runtime, memory usage, and CPU efficiency. We also highlighted their suitability for different research needs, from analyses focused on speed, like those enabled by SUPPA2, to more comprehensive, resource‐intensive tools like MISO and Cufflinks.

Our analysis covered various aspects of each tool's functionality. For example, we examined how each tool processes input data, the types of AS events they can detect, and their compatibility with different sequencing technologies. We also investigated how well these tools scale with increasing dataset sizes and their performance in terms of runtime and memory consumption. Additionally, we assessed CPU efficiency to understand how these tools utilize computational resources. By presenting a detailed and standardized review, we aimed to provide a clear picture of each tool's capabilities and limitations. This information is crucial for researchers who need to choose the most appropriate AS detection tool based on their specific requirements. For instance, researchers conducting large‐scale analyses may prioritize tools that offer high speed and scalability, such as SUPPA2. In contrast, those requiring more precise splicing event detection might opt for more resource‐intensive tools like MISO and Cufflinks, which offer comprehensive analysis at the cost of increased computational demands. We hope this information facilitates the development of next‐generation AS detection tools.

By identifying current challenges, such as scalability, computational efficiency, and ease of implementation, we hope to guide future efforts in creating more advanced and user‐friendly tools. We hope this review helps researchers navigate the complex landscape of AS detection and select the best tools for their specific research needs.

## Author Contributions

**Hieu Tran:** conceptualization (equal), formal analysis (equal), investigation (lead), methodology (lead), writing – original draft (equal). **Nirad Banskota:** data curation (equal), formal analysis (equal), investigation (equal), methodology (equal), writing – original draft (equal). **Myriam Gorospe:** conceptualization (supporting), visualization (supporting), writing – review and editing (lead). **Supriyo De:** conceptualization (equal), investigation (supporting), supervision (lead), writing – original draft (equal), writing – review and editing (supporting).

## Conflicts of Interest

The authors declare no conflicts of interest.

## Related WIREs Articles

Integrated Biochemical and Computational Methods for Deciphering RNA‐Processing Codes

Alternative Splicing and CaV‐Associated Channelopathies

## Data Availability

Data sharing is not applicable to this article as no new data were created or analyzed in this study.
